# Integrated omics profiling of individual variations in intestinal damage to the soybean allergen in piglets

**DOI:** 10.3389/fvets.2024.1521544

**Published:** 2025-01-15

**Authors:** Mengmeng Mi, Yaqing Zheng, Xin Fu, Nan Bao, Li Pan, Guixin Qin, Yuan Zhao

**Affiliations:** Key Laboratory of Animal Production, Product Quality and Security, Ministry of Education, Jilin Provincial Key Laboratory of Animal Nutrition and Feed Science, College of Animal Science and Technology, Jilin Agricultural University, Changchun, China

**Keywords:** weaned piglets, soybean antigen, β-conglycinin, intestine damage, omics profiling

## Abstract

**Introduction:**

A small number of soybean allergens [including Glycinin (11S) and β-Conglycinin (7S)] in the commercially available corn-soybean meal diet can still cause allergy in some weaned piglets, which may be the result of the interaction of genetic, and nutrition, but the specific mechanism is still unclear.

**Methods:**

In this study, 20 allergic piglets and 20 non-allergic piglets were selected from 92 weaned piglets by skin sensitization tests, which were used to examine the whole sequence genome. The indicators related to humoral and cellular immunity, transcriptomics, and metabolomics analysis were determined by randomly selecting 5 boars in the allergic group and non-allergic group and then performing a validation *in vitro*.

**Results:**

The sensitization rate of soybean antigen in the corn-soybean meal diet was 21.74% and there was a gender difference with the sensitization rate of female pigs (31.34%) being higher than that of male pigs (13.23%). Moreover, the levels of inflammatory factors (IL-1β, IL-4, TNF-α) and antibodies (IgG, IgE, and specific IgG) in allergic piglets were significantly higher than those in non-allergic piglets (*P* < 0.05). Whole genome re-sequencing analysis revealed specific mutations in the exons and URT5 of TRAPPC2, PIR, CFP, and SOWAHD genes and showed significantly higher expression levels of related genes in the spleen of allergic piglets (*P* < 0.05). Transcriptome analysis identified IL17REL, CCL19, CD1E, CD1.1, etc. immune differential genes, metabolomics results showed that soybean antigen affected the utilization and metabolism of intestinal nutrients in piglets, mainly the digestion and absorption of protein and the synthesis and metabolism of amino acids. Transfection of CFP/TRAPPC2/CCL19 siRNA could partially alleviate the injury of RAW264.7 cells or IPEC-J2 cells induced by β-Conglycinin.

**Conclusion:**

Therefore, the individual differences in intestinal damage induced by soybean antigen protein in the corn-soybean meal diet are closely related to PIR, CFP, TRAPPC2, SOWAHD, and CCL19 genes. Soybean antigens affect the intestinal nutrient utilization and metabolism of piglets, which provides a scientific reference for the study of soybean antigen sensitization mechanisms, precision nutrition, disease prevention, and control of piglets, and also lays a foundation for human foodborne diseases.

## 1 Introduction

Soybean, a high-quality plant protein source for humans and livestock, is widely used in human food and animal feed. Soybean antigen, as an anti-nutritional factor with thermal stability, can change the morphology and damage the structure of small intestine tissues (1), and induce autophagy (2), and apoptosis (3) in small intestinal epithelial cells (4), and also negatively affect the content and composition of intestinal metabolites (5, 6), leading to digestion and absorption disorders, growth retardation and allergic diarrhea (7). Nevertheless, the immune-pathological mechanism of soybean allergy remains unclear and requires further exploration.

There is heterogeneity inherent in the anaphylaxis amongst allergen-sensitized individuals (8). Soybean antigens can induce allergy in piglets (9), calves (10), and rats (11), but piglets had a stronger sensitivity to β-conglycinin than the other animal species and presented different β-conglycinin-specific epitopes (12). Furthermore, a distinct sensitivity to soybeans was observed between breeds of pigs. For example, the sensitivity of Min pigs to soybeans was much lower than that of Landrace pigs (13), which might be ascribed to the genetic differences (8, 14, 15). Studies have suggested that immune genes such as MHC-I (16), IL-1, IL-10 (17), IL-17 (18) may be the genetic basis for allergic reactions. Additionally, gender may also play a role in the incidence of food allergies (19–21). Also, it was found that female mice are more susceptible to allergies (22). Therefore, there is a need to investigate the reasons for individual varied reactions to soybean allergens.

Soybean meal in a standard formula diet with the conventional procession still retains a small amount of soybean allergens that would cause transient hypersensitivity reactions in piglets, especially at the early weaning stage (23), but related research is scanty. In this study, a nutrigenomics-based multidimensional approach was utilized to determine the individual heterogeneity of soybean-induced allergy in weaned piglets fed continuously with corn-soybean meal diets. Then, based on the experimental results of variances of genomic resequencing, transcriptome, and metabolites between allergic piglets and non-allergic piglets, this study would provide a theoretical foundation for the mechanism of soybean allergy, and also better the precise nutrition and breeding of pigs.

## 2 Materials and methods

### 2.1 Animals

The animal experiment was approved by the Jilin Agricultural University Animal Care and Use Committee. In Hua Zheng pig farm, the 92 Landrace × Yorkshire piglets weaned at 27 days were obtained as the population. Based on the report measured the content of soybean antigen in the diet by competitive ELISA (24), containing 2.53% Glycinin and 1.99% β-Conglycinin. Piglets were fed a soybean diet for 7 days had free access to water and were all housed under the same environmental conditions. First, 20 allergic and 20 non-allergic piglets were differentiated by using the skin sensitization test, and then randomly selected from the 92 piglets for whole genome resequencing analysis. Second, from this group, 5 allergic and 5 non-allergic were randomly chosen from the 20 allergic and 20 non-allergic piglets for the follow-up experiments with the samples of serum and jejunum.

### 2.2 Skin sensitization test

The skin sensitization test was conducted to judge the sensitive response of the piglets to soybean allergens, the doses of Glycinin and β-Conglycinin in skin tests were referred to in the previous study (12). In detail, a 0.5 mL allergic protein (purified Glycinin or β-Conglycinin) in 0.9% sodium chloride solution was intradermally injected into the abdominal skin of piglets, and 0.5 mL 0.9% sodium chloride solution with the same volume injected at the control site. Thirty minutes after injection, the erythema diameters were measured using a vernier caliper. An erythema diameter of over 5 mm was regarded as an allergy.

The ear tissues from screened piglets, including 20 allergies and 20 non-allergies, were collected to analyze the whole-genome sequence. Five piglets, respectively, from the allergic or non-allergic group were selected and slaughtered, and their serum and jejunum tissue were collected to detect the immune indices, gene expression, and metabolites.

### 2.3 Analysis of serum biomarkers

The contents of total protein (TP), albumin (ALB), globulin (GLB), and alkaline phosphatase (AP) were determined by an automatic biochemical analyzer, and then the ratio of albumin and globulin (A/G) was calculated.

### 2.4 Analysis of cytokine levels by ELISA

Concentrations of interleukin 1-β (IL-1β), IL-4, interferon-γ (IFN-γ), and tumor necrosis factor-α (TNF-α) in serum were determined using the swine ELISA kit according to the manufacturer's instructions.

### 2.5 Analysis of total IgG, IgE, IgM, sIgA in serum and mucosa and specific IgG levels by ELISA

Total IgG, IgE, IgM, and sIgA levels in serum and mucosa were determined by using swine ELISA kits according to the manufacturer's instructions. Glycinin-specific IgG and β-Conglycinin-specific IgG antibody levels were detected via an indirect ELISA as described by Sun et al. (25).

### 2.6 Variant detection by resequencing

Genomic DNA was extracted from ear tissues using a 2% CTAB-isopropyl alcohol precipitation, and DNA was detected using 0.8% agarose gels. TruSeq DNA PCR-free prep Kit (Paiseno Biotech Co., Ltd., Shanghai, China) was utilized to construct a sequencing library with an insert size of 400 bp, a representative library was built by each individual, and sequencing library quality inspection was performed using the Agilent High Sensitivity DNA Kit (Paiseno Biotech Co., Ltd., Shanghai, China). The Quant-iT PicoGreen dsDNA Assay Kit (Paiseno Biotech Co., Ltd., Shanghai, China) was used to quantify the library on the Paiseno Quanti Fluor fluorescence quantification system. High-quality data were obtained after raw data evaluation and filtering. The dataset was subsequently compared with the reference genome (Sscrofa 11.1) using BWA-MEM (0.7.12-r1039) (26). Duplicates were removed through the “MarkDuplicates” of the Picard software package to improve the accuracy of SNP prediction. GATK (27) and ANNOVAR (28) were used to detect and annotate SNPs and InDels (29).

### 2.7 Total RNA extraction and transcriptome analysis

Jejunal tissues were collected for RNA sequencing and transcriptomics, and the RNA extraction and sequencing transcriptomics were performed by Shanghai Personal Biotechnology Co., Itd. (Shanghai, China). Total RNA was extracted from the intestine samples and purified using the Trizol Reagent (Invitrogen, Carlsbad, CA) and the RNeasy mini kit (Qiagen, Venlo, Netherlands). Thereafter, RNA purity and integrity were assessed using the Nanodrop spectrophotometer (Thermo Scientific, USA) and the Agilent High Sensitivity RNA Kit of the Agilent 2100 Bioanalyzer (Agilent Technologies, CA, USA), and RNA library preparation was realized using the Illumina TruSeq Stranded Total RNA kit. Moreover, paired-end sequencing was performed on Illumina HiSeq X Ten, and transcriptomic data were aligned and mapped onto the complete genome of the reference genome (Sscrofa 11.1). Furthermore, differential gene expression analysis was normalized to reads per kilobases per million reads (RPKM) values, and the differential expressions of the genes were analyzed by DESeq (1.30.0) with screened conditions as follows: expression difference multiple |log2FoldChange| > 1, *P* < 0.05. Then all the genes to Terms in the Gene Ontology database, and to carry out the enrichment analysis of the KEGG pathway of differential genes, focusing on the significant enrichment pathway with *P* < 0.05.

### 2.8 Identification of metabolites and metabolomics analysis

The metabolites in the jejunum sample were detected via gas chromatography-mass spectrometry (GC-MS). Take about 100 mg of jejunum sample and add 1 mL of pre-cooled methanol: acetonitrile: Water (2:2:1, v/v), homogenate crushing (24 × 2, 6.0 M/S, the 20 s, 3 times) with MP homogenizer, stand at −20°C for 60 min, centrifuge at 13,000 g at 4°C for 15 min, supernatant (divided into 900 μL/tube), vacuum drying, The freeze-dried powder was stored at −80°C for use; during mass spectrum analysis, 100 μL acetonitrile aqueous solution (acetonitrile: water = 1:1, v/v) was added for resolution, vortex, then 14,000 g at 4°C for 15 min, and the supernatant was taken for metabolomic analysis. The supernatants were separated by Agilent 1290 Infinity LC ultra-high performance Liquid chromatography (UHPLC) HILIC column and detected by electrospray ionization (ESI) positive and negative ion modes, respectively. Then, the differential metabolites were identified based on variable importance in projection (VIP) ≥ 1 (generated by the OPLS-DA model) and *P* ≤ 0.05. Differential metabolites were mapped into the Kyoto Encyclopedia of Genes and Genomes (KEGG) pathway database to analyze their metabolic pathway.

### 2.9 Cytokine levels of β-conglycinin-induced RAW264.7 cells and IPEC-J2 cells treated with effective siRNA

For further investigation of the relationship between candidate genes and β-Conglycinin, the siRNA technique employed in this study used cells. The RAW264.7 cells and IPEC-J2 cells were purchased from Shanghai EK-Bioscience Biotechnology Co., Ltd. The cells were inoculated in cell culture bottles and cultured in a DMEM/high glucose medium (Hyclone, USA) or DMEM/F12 medium (Hyclone, USA). The porcine jejunal cell line IPEC-J2 is a good model for studying intestinal health and the dose of β-Conglycinin in IPEC-J2 cell was referred to as Mi et al. (30), and the RAW264.7 cell is the main cell model for immunological research (31), and pre-experimentally screened the dose of RAW264.7 cells treated with β-Conglycinin. The cell culture and cell viability assay were conducted performed by adopting the methods Mi et al. (30), and Yi et al. (31).

The gene silencing experiment was divided into control (untreated cells), negative control (NC), 7S group (β-Conglycinin (7S)-treated cells), siCFP group (siCFP-treated cells), and 7S + siRNA group (7S + siCFP-treated cells). After being diluted in opti-MEM Medium (Invitrogen), siRNAs AND Lipofectamine were mixed and conducted following the Lipofectamine RNAi MAX Transfection Reagent Handbook (Invitrogen, USA), and siRNAs were transferred into RAW264.7 and IPEC-J2 cells at 37°C for 24 h. According to the instructions of the manufacturer, the concentrations of IL-1β, IL-6, IL-10, and TNF-α from RAW264.7 cells, and the levels of IL-6, IL-10, TNF-α, NF-κB, IFN-γ from IPEC-J2 cells were measured. Additionally, the relative mRNA expression level of related cytokines was determined by the qRT-PCR.

### 2.10 Qualitative real-time PCR (qRT-PCR)

Total RNA content was isolated from the cells, and the spleen of the allergic group and non-allergic group piglets using TRIzol reagent (Takara, Japan). After the DNase treatment, the RNA was reverse-transcribed to single-stranded cDNA using a Prime Script RT reagent kit (Takara, Japan, RR047A), and the stable housekeeping gene was used as the control. Quantitative real-time PCR (qRT-PCR) was performed using SYBR Green Premix Ex Taq (TRAN, AQ601; Takara, Japan, RR820A) on a StepOnePlus^TM^ real-time PCR Detection System (BioRad, Hercules, CA, USA). The qRT-PCR primer pairs were observed for five genes (Supplementary Table 1). Using the comparative CT method (2^−ΔΔ*CT*^ method, (ΔΔ*CT* = Δ*CT*(treated gene)−Δ*CT*(control), Δ*CT* = *CT*(target gene)−*CT*(reference gene))) was used to analyze the transcription levels of different genes.

### 2.11 Statistical analysis

Student's *t*-test was used to compare the data between the two groups, and data among three or more groups were analyzed with ANOVA followed by Duncan's multiple comparison test. SPSS 23.0 software (IBM Inc., Armonk, NY, USA). Data were presented as mean ± standard deviation. Differences were considered statistically significant when *P* < 0.05.

## 3 Results

### 3.1 Cutaneous hypersensitivity reactions

**Figure 1:**
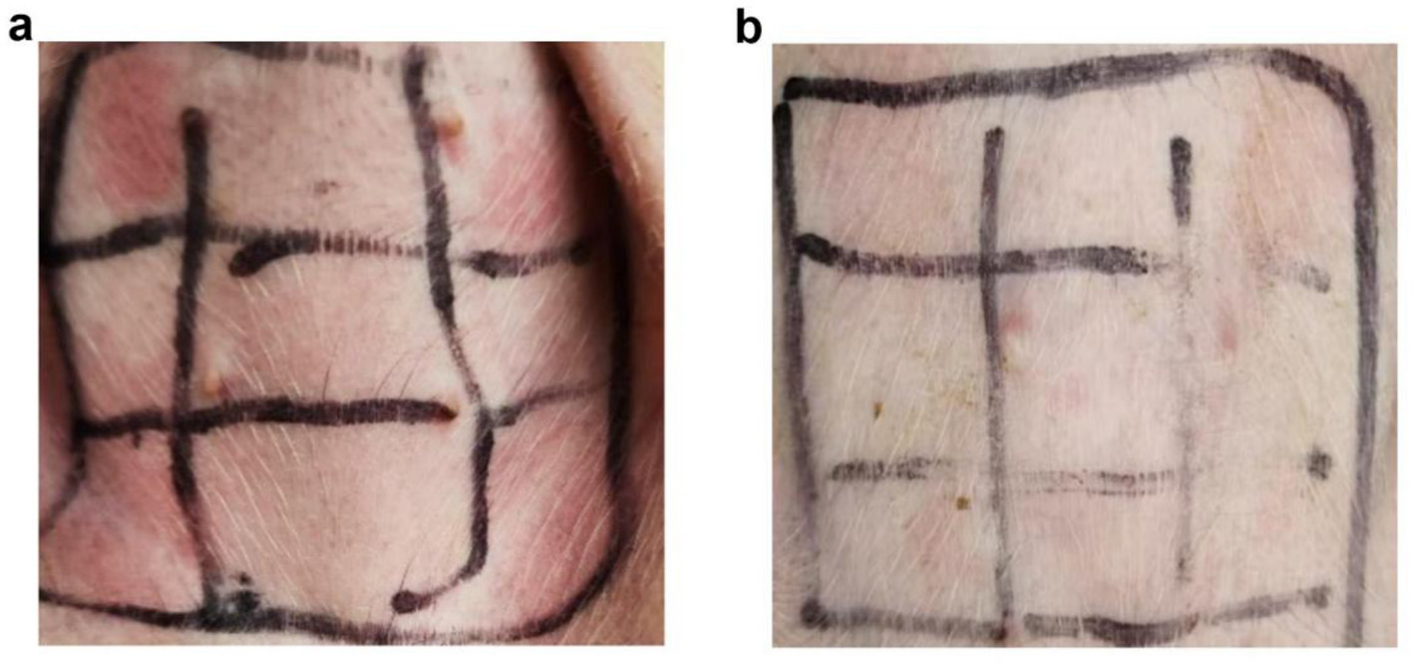
Results of skin prick test in piglets. **(A)** Allergic group; **(B)** Non-allergic group.

In the study, a typical skin response was observed in Figure 1A, while no significant change was observed in non-allergic piglets, as shown in Figure 1B. The erythema diameters in both allergic and non-allergic groups were recorded in Supplementary Table 2. Out of the 92 weaned piglets studied, 21.74% were found to have soybean-induced allergies. Interestingly, the allergy rate was higher in female piglets (31.34%) than in male piglets (13.23%).

### 3.2 The serum biochemical indices levels

**Figure 2:**
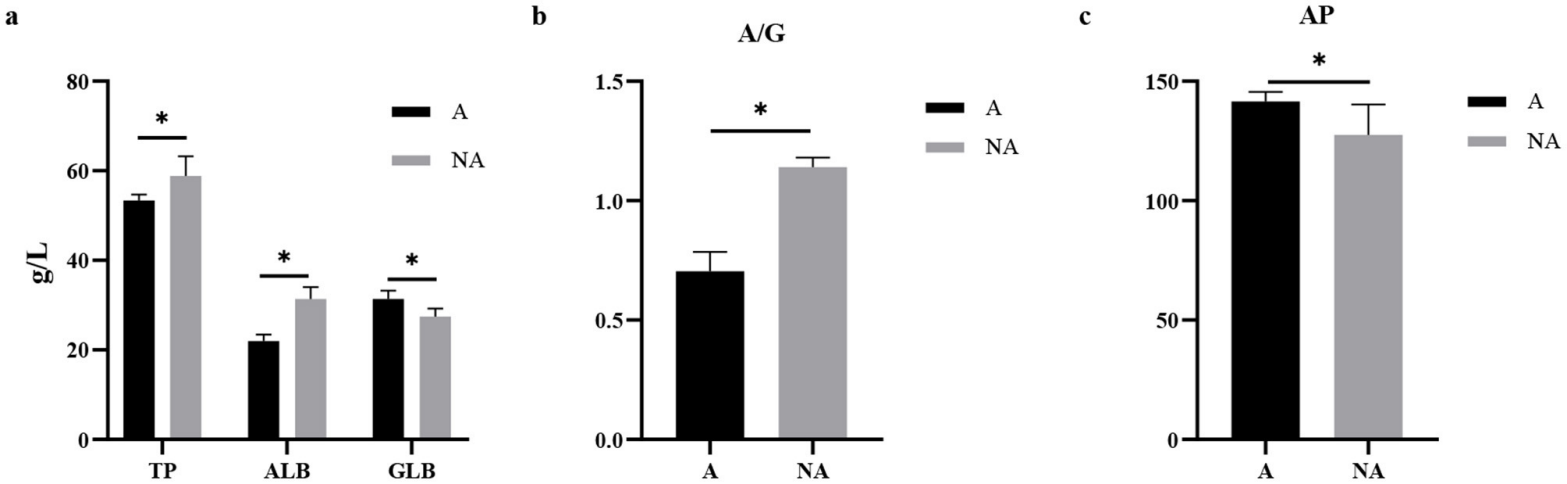
The levels of biochemical indicators in serum. A, Allergic group; NA, Non-allergic group. **(A–C)** showed the levels of TP, ALB, GLB, A/G, and AP in serum. *P < 0.05.

Compared with the non-allergic group, in serum, the levels of TP and ALB and the A/G ratio in the allergic group were significantly decreased (*P* < 0.05), meanwhile, the serum AP and globulin (GLB) levels of piglets in the allergic group were significantly higher than those in the non-allergic group (*P* < 0.05) (Figure 2). Our study found that piglets with allergies had higher GLB and AP levels, and lower ALB levels, indicating that inflammation response enhanced and impaired intestinal function in the piglets.

### 3.3 The serum inflammation cytokine level

**Figure 3:**
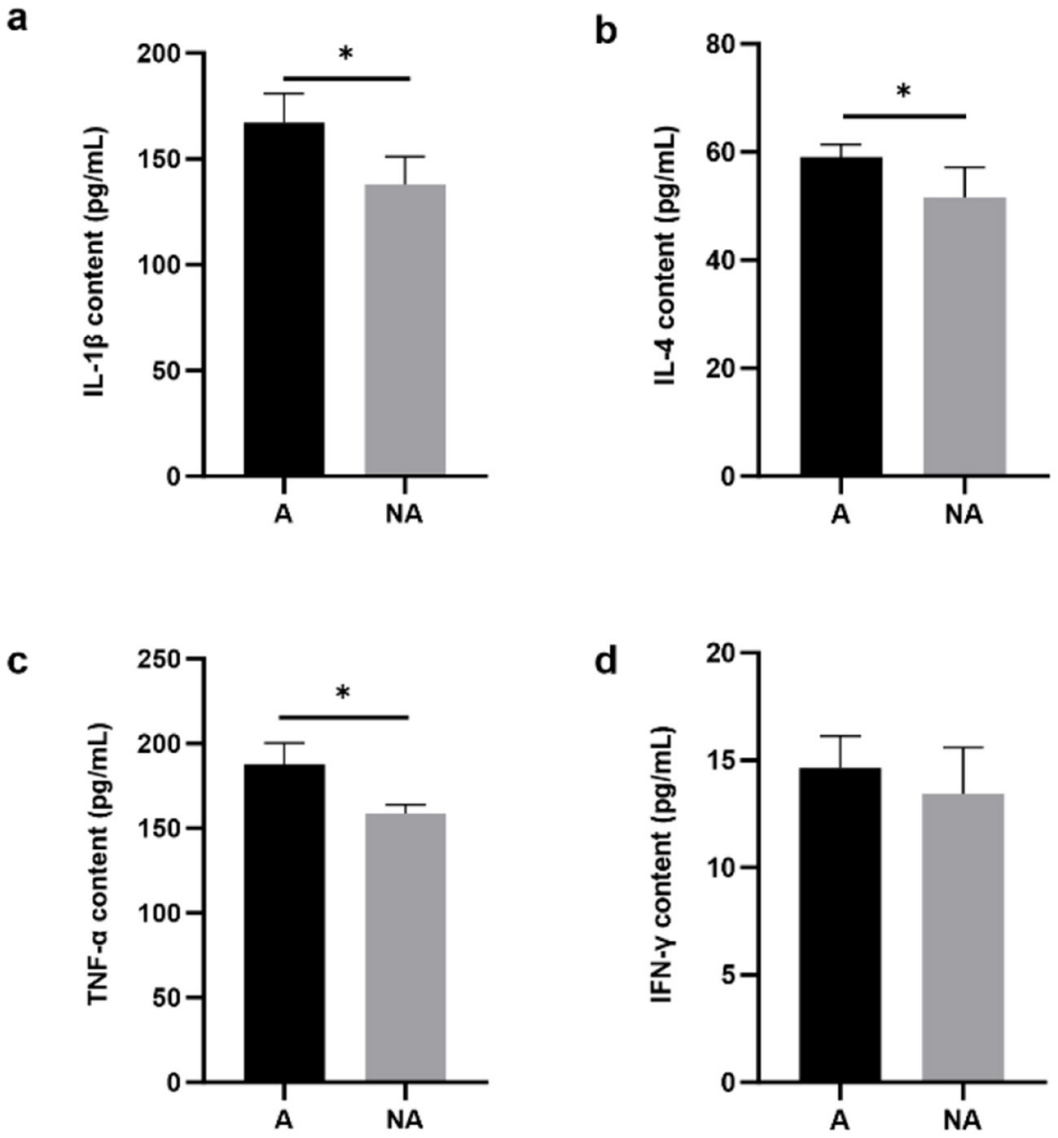
The levels of inflammatory factors in serum. A, Allergic group; NA, Non-allergic group. **(A–D)** showed the levels of IL-1β, IL-4, TNF-α, and IFN-γ in serum. *P < 0.05.

The results showed that in the serum, the IL-4, TNF-α, and IL-1β levels were significantly higher in the allergic group (*P* < 0.05), whereas there was no significant difference in the level of IFN-γ (*P* = 0.334) between the two groups (Figure 3). This indicated that soybean antigens increased inflammatory factor levels, and caused inflammation damage in piglets.

### 3.4 Total serum and mucosa IgG, IgE, IgM, and sIgA and specific IgG levels

**Figure 4:**
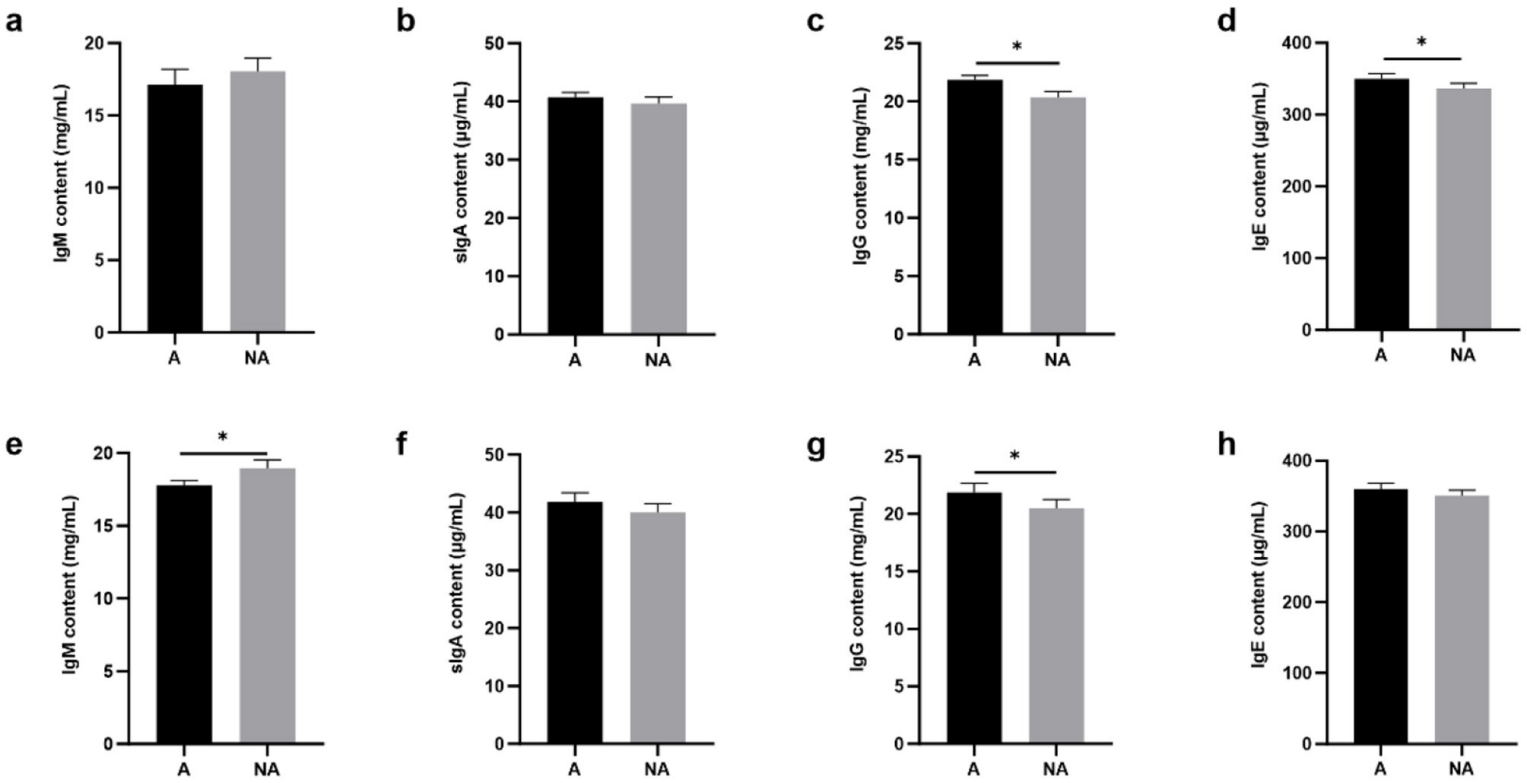
The levels of total antibody in serum and mucosal. A, Allergic group; NA, Non-allergic group. **(A–D)** showed that the levels of IgM, sIgA, IgG, IgE in serum; **(E–H)** showed that the levels of IgM, sIgA, IgG, IgE in the mucosa. *P < 0.05.

**Figure 5:**
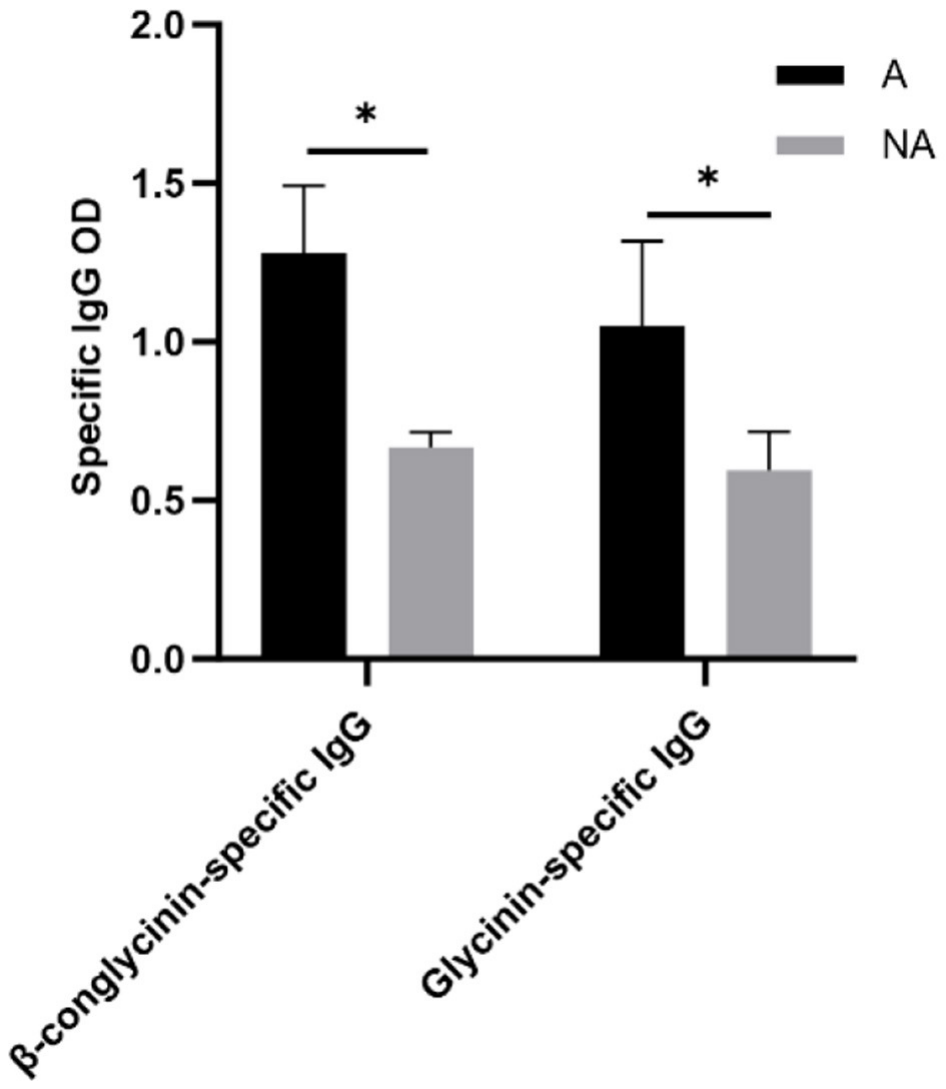
The levels of Glycinin and β-conglycinin-specific IgG in serum. A, Allergic group; NA, Non-allergic group. *P < 0.05.

To explore the effects of soybean-induced allergy in sensitized pigs, the total serum and mucosa IgG, IgE, IgM, and sIgA levels in serum were determined (Figure 4). The total serum IgG and IgE were significantly higher in the allergic group (*P* < 0.05). In the mucosa, the non-allergic group had a higher IgM (*P* < 0.05), and the IgG level significantly decreased compared with the allergic group (*P* < 0.05), and the IgE level was some higher but not significantly in the allergic group than in the non-allergic-group (*P* = 0.093). The specific IgG levels are shown in Figure 5, Glycinin-specific IgG and β-conglycinin-specific IgG were significantly higher in the allergic group compared with the non-allergic group (*P* < 0.05). This indicated that soybean antigens increased immunoglobulin levels, and induced the immune response.

### 3.5 Functional clustering of mutant genes

To clarify the genetic mechanisms involved in regulating the soybean antigen on pigs by the whole genomic resequencing. In Table 1, at GA and GNA groups, there are a total number of 28 specific Indels located in 4 genes, in which there were 21 Indels mutations in the UTR5 of the PIR and CFP genes in the GA group, and there were 2 Indels mutations in the exon which had the frameshift deletion in the LOC110257895 gene in GNA group.

**Table 1:** The functional Indels on specific genes.

**Group**	**Gene**	**Region**	**Mutation-type**	**Chrome**	**ref**	**alt**
GA	PIR	UTR5		X	T	T
GA	PIR	UTR5		X	T	G
GA	PIR	UTR5		X	T	C
GA	PIR	UTR5		X	T	C
GA	PIR	UTR5		X	T	C
GA	PIR	UTR5		X	T	T
GA	PIR	UTR5		X	T	T
GA	PIR	UTR5		X	T	G
GA	PIR	UTR5		X	T	A
GA	PIR	UTR5		X	T	C
GA	PIR	UTR5		X	T	C
GA	PIR	UTR5		X	T	A
GA	PIR	UTR5		X	T	T
GA	PIR	UTR5		X	T	A
GA	PIR	UTR5		X	T	T
GA	PIR	UTR5		X	T	G
GA	CFP	UTR5		X	G	G
GA	CFP	UTR5		X	G	G
GA	CFP	UTR5		X	G	G
GA	CFP	UTR5		X	G	G
GA	CFP	UTR5		X	T	G
GA	NDUFA1	Downstream		X	C	C
GA	NDUFA1	Downstream		X	C	C
GA	NDUFA1	Downstream		X	C	T
GA	NDUFA1	Downstream		X	C	C
GA	NDUFA1	Downstream		X	C	A
GNA	LOC110257895	Exonic	Frameshift deletion	Y	AG	A
GNA	LOC110257895	Exonic	Frameshift deletion	Y	G	A

GA, Genomic in the allergic group; GNA, Genomic in the non-allergic group.

In Table 2, at GA and GNA groups, there are a total number of 4 specific SNPs located in the exon of 2 genes, of which 4 non-synonymous mutations were located in 2 genes. A total number of 11 special SNPs were located in the UTR5 of 4 genes. At the genomic level, there was only one specific SNP located in the non-synonymous mutation in the GA group, and in the GNA group, there were three specific SNPs in the non-synonymous mutations. The specific SNPs of allergy and non-allergic groups were mainly in trafficking protein particle complex subunit 2 (TRAPPC2), Pirin (PIR), complement factor properdin (CFP), and sosondowah ankyrin repeat domain family member D (SOWAHD), however only TRAPPC2 and PIR were non-synonymous mutations. We also found changes in amino acids located in genes and changes in bases at mutation sites.

**Table 2:** The functional SNPs on specific genes.

**Group**	**Gene**	**Region**	**Mutation-type**	**AA mutation**	**Chrome**	**ref-alt**	**alt**
GA	PIR	Exonic	Non-synonymous	M8T	X	A	G
GA	PIR	UTR5			X	C	A
GA	PIR	UTR5			X	A	G
GA	PIR	UTR5			X	C	A
GA	PIR	UTR5			X	A	T
GA	PIR	UTR5			X	G	A
GA	PIR	UTR5			X	A	T
GA	PIR	UTR5			X	C	T
GA	PIR	UTR5			X	T	C
GA	CFP	UTR5			X	T	G
GA	SOWAHD	UTR5			X	C	T
GNA	TRAPPC2	Exonic	Non-synonymous	E21K	X	C	T
GNA	TRAPPC2	Exonic	Non-synonymous	F18L	X	A	C
GNA	TRAPPC2	Exonic	Non-synonymous	M1T	X	C	G
GNA	TRAPPC2	UTR5			X	C	T

GA, Genomic in the allergic group; GNA, Genomic in the non-allergic group; (M8T), The 8th amino acid is mutated from methionine to threonine; (E21K), The 21st amino acid is mutated from glutamate to lysine; (F18L), The 18th amino acid is mutated from phenylalanine to leucine; (M1T), The first amino acid was mutated from methionine to isoleucine.

### 3.6 Transcriptomic analysis

**Figure 6:**
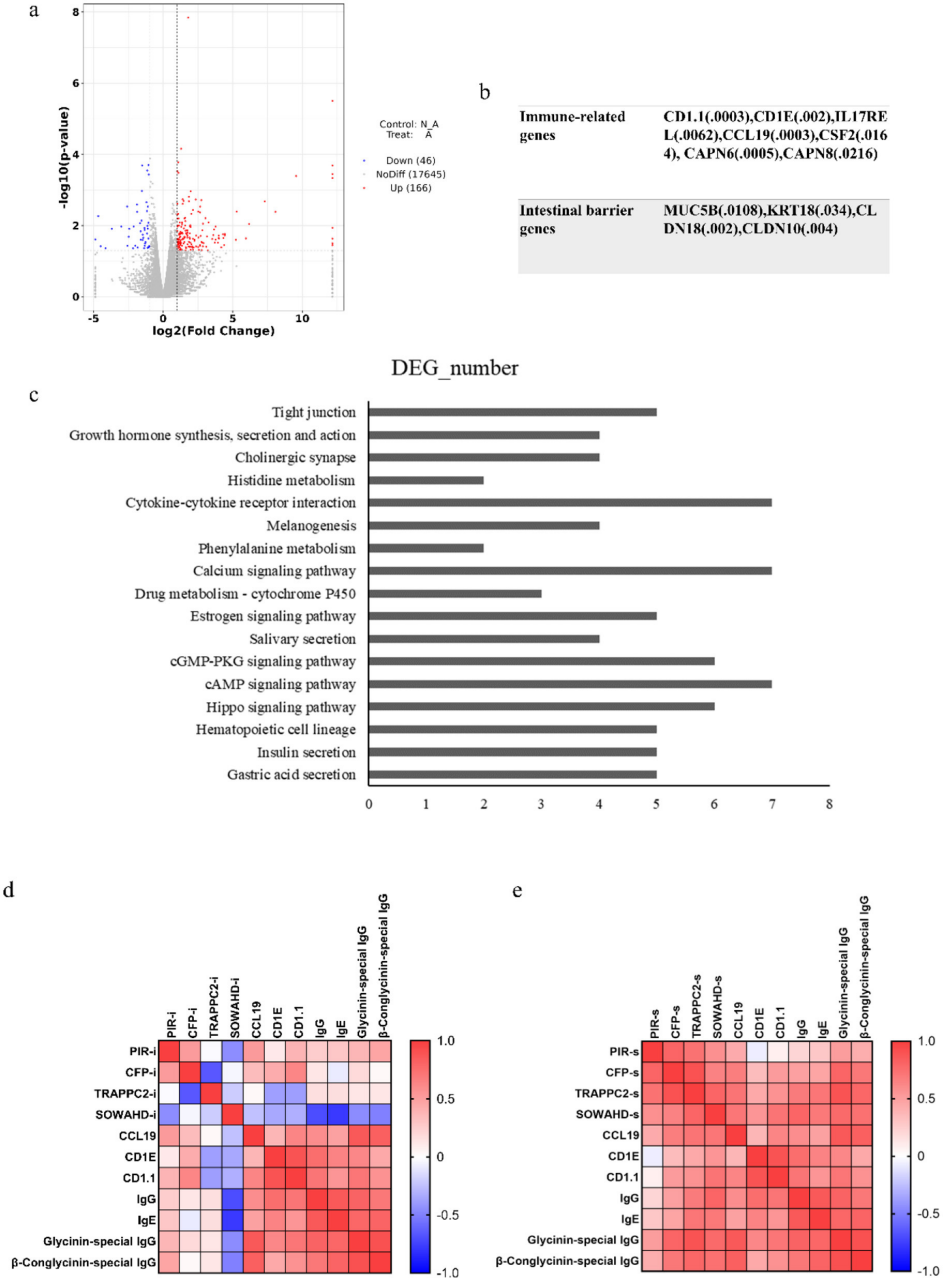
Intestinal transcriptomic analysis. **(A)** Statistics of differential gene expression results; **(B)** The genes related to immune and intestinal barrier; **(C)** Differential genes at KEGG pathway (top 20); Correlation analysis between candidate genes in intestine **(D)** or spleen **(E)** and immune responses. *P* < 0.05.

In Figure 6, the initial statistical analyses of the transcriptome data showed that a total of 212 genes were differentially expressed, including 166 up-regulated and 46 down-regulated genes (Figure 6A). In the allergic group, soybean antigen increased the expression of immune-related genes such as CD1.1, CD1E, IL17REL, CCL19, CSF2, CAPN6, CAPN8, and IL1R2 and increased the expression of genes related to intestinal barrier such as MUC5B, KRT18, CLDN18 and CLDN10 (*P* < 0.05) (Figure 6B). The largest quantity of differential expression genes (DEGs) was classified into the “cAMP signaling pathway,” “Calcium signaling pathway,” and “Cytokine-cytokine receptor interaction.” Moreover, genes involved in Insulin secretion, the Estrogen signaling pathway, the Drug metabolism-cytochrome P450 pathway, Phenylalanine metabolism, Histidine metabolism, Throsine metabolism, Glycine, serine and threonine metabolism were also differentially regulated (Figure 6C), indicating these pathways were part of the piglet response to soybean antigens.

Transcriptome sequencing results showed that there were differences in the transcriptional levels of PIR, CFP, TRAPPC2, and SOWAHD genes in the intestinal tract of allergic and non-allergic piglets. Among them, the relative expression levels of PIR and CFP genes in the intestines of allergic piglets were higher than those of non-allergic piglets (Supplementary Table 3). The expression of PIR and CFP genes in the intestine and spleen was positively correlated with the level of immune-related genes in the intestine and the antibody in the serum, indicating that the expression of PIR and CFP genes could promote the immune response. However, the transcription levels of SOWAHD and TRAPPC2 genes in the intestine have no up-trend and are positively correlated with immune-related genes and antibody levels when expressed in the spleen, which may be related to their expression site or technical error (Figures 6D, E).

### 3.7 Metabonomic analysis

**Figure 7:**
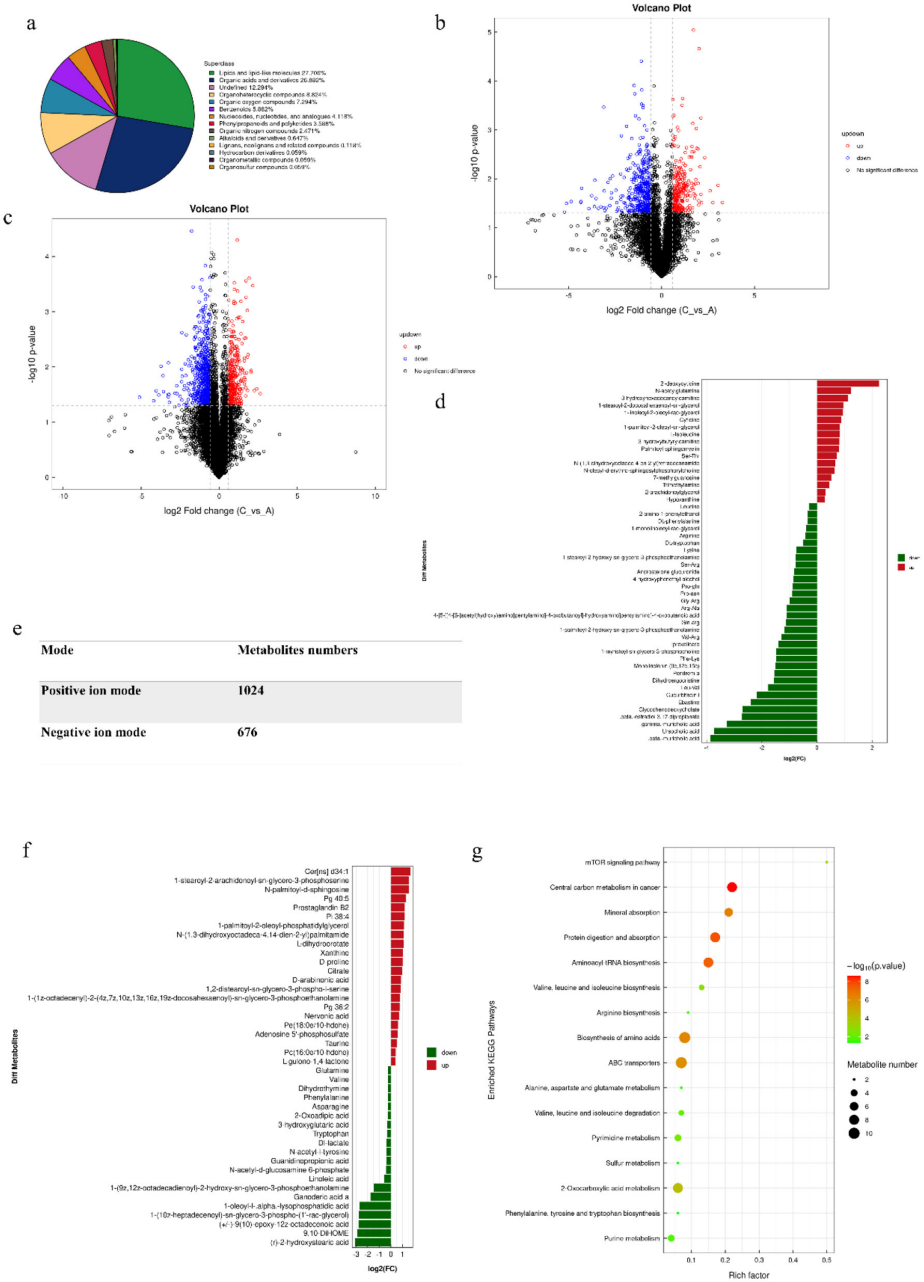
The results with metabolomics analysis. **(A)** Chemical classification and attribution statistics of metabolites; **(B)** Negative ion mode volcano map; **(C)** Positive ion mode volcano map; **(E)** Statistics of the number of metabolites identified in the positive and negative ion mode; **(D)** The differential metabolites in positive ion mode; **(F)** The differential metabolites in negative ion mode volcano map; **(G)** The differential metabolites pathway.

The metabolic profiles of the intestine samples between allergic and non-allergic groups were detected using untargeted metabolomics analysis (Figure 7). Initially, we found that the most important metabolites were lipid and lipid-like molecules, accounting for 27.706% of all metabolites. Organic acids and their derivatives followed closely behind, accounting for 26.882%. We discovered that 17 metabolites were significantly up-regulated and 33 metabolites were significantly down-regulated in positive mode. In negative mode, 20 metabolites were significantly up-regulated and 22 metabolites were significantly down-regulated. Some metabolites, such as phenylalanine, tryptophan, leucine, arginine, valine, glutamine, and hypoxanthine, were significantly increased in the allergic group (*P* < 0.05). The analysis of KEGG pathways showed that differential metabolites were enriched in the pathways of Central carbon metabolism in cancer, Protein digestion and absorption, Aminoacyl-tRNA biosynthesis, Biosynthesis of amino acids, ABC transporters, mTOR signaling pathway, Valine, leucine and isoleucine biosynthesis, and Pyrimidine metabolism. Most of the metabolites were enriched in the pathway of protein digestion and absorption, and biosynthesis of amino acid, suggesting that soybean antigen could affect the metabolism and utilization of nutrients in the intestine of piglets.

The correlation analysis results showed that the synthesis and metabolism of amino acids were closely related to the body's immune function, while the pyrimidine metabolism pathway was negatively correlated with the immune ability (Supplementary Table 4).

### 3.8 Expression levels of the four specific genes in the spleen

**Figure 8:**
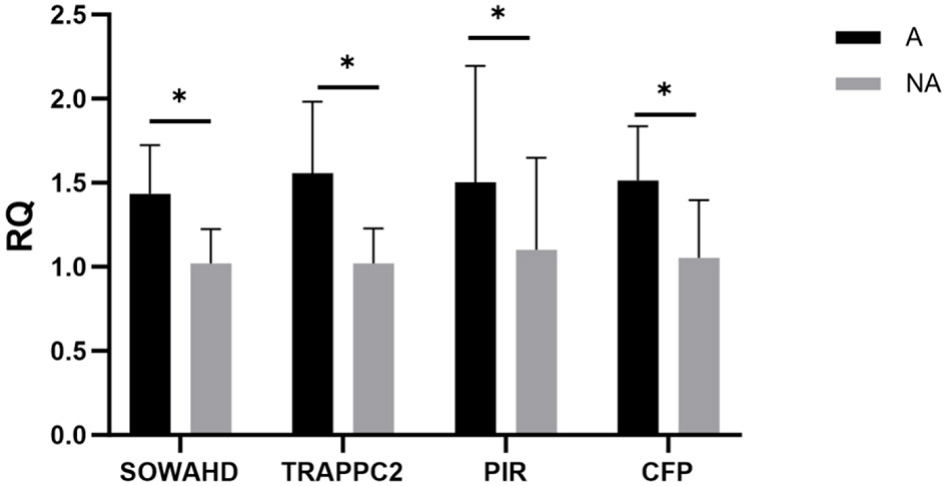
The expression levels of different genes. A, Allergic group; NA, Non-allergic group. *P < 0.05.

To verify candidate genes for the whole-genome re-sequencing, the relative expression levels of related genes in the spleen of piglets in the allergic and non-allergic groups were analyzed by qualitative real-time PCR. As shown in Figure 8, the expression levels of the SOWAHD, TRAPPC2, PIR, and CFP in the allergic group were significantly higher than those in the non-allergic group (*P* < 0.05).

### 3.9 Effects of the cytokine levels of β-conglycinin (7s)-induced RAW264.7 cells treated with effective CFP siRNA or TRAPPC2 siRNA

**Figure 9:**
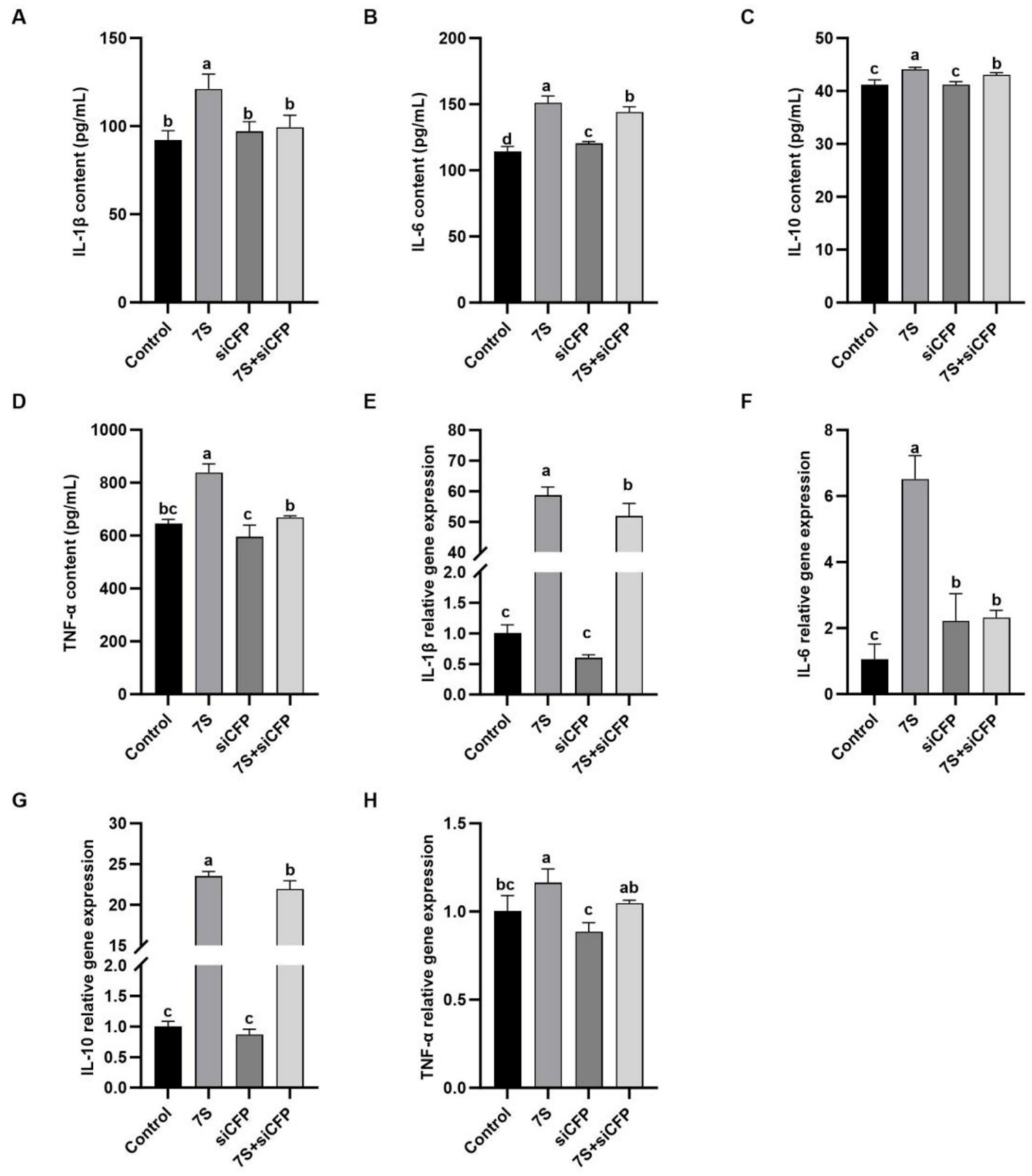
Effects of the cytokine levels of β-Conglycinin-induced RAW264.7 cells treated with effective CFP siRNA. Control: untreated cells; 7S group: β-Conglycinin (7S)-treated cells; siCFP group: siCFP-treated cells; 7S + siCFP group: 7S + siCFP-treated cells. **(A–D)** The levels of inflammatory factor content (IL-1β, IL-6, IL-10, TNF-α) in the RAW264.7 cells supernatants. **(E–H)** The levels of inflammatory factor expression (IL-1β, IL-6, IL-10, TNF-α) in the RAW264.7 cells. ^a, b, c^*P* < 0.05.

**Figure 10:**
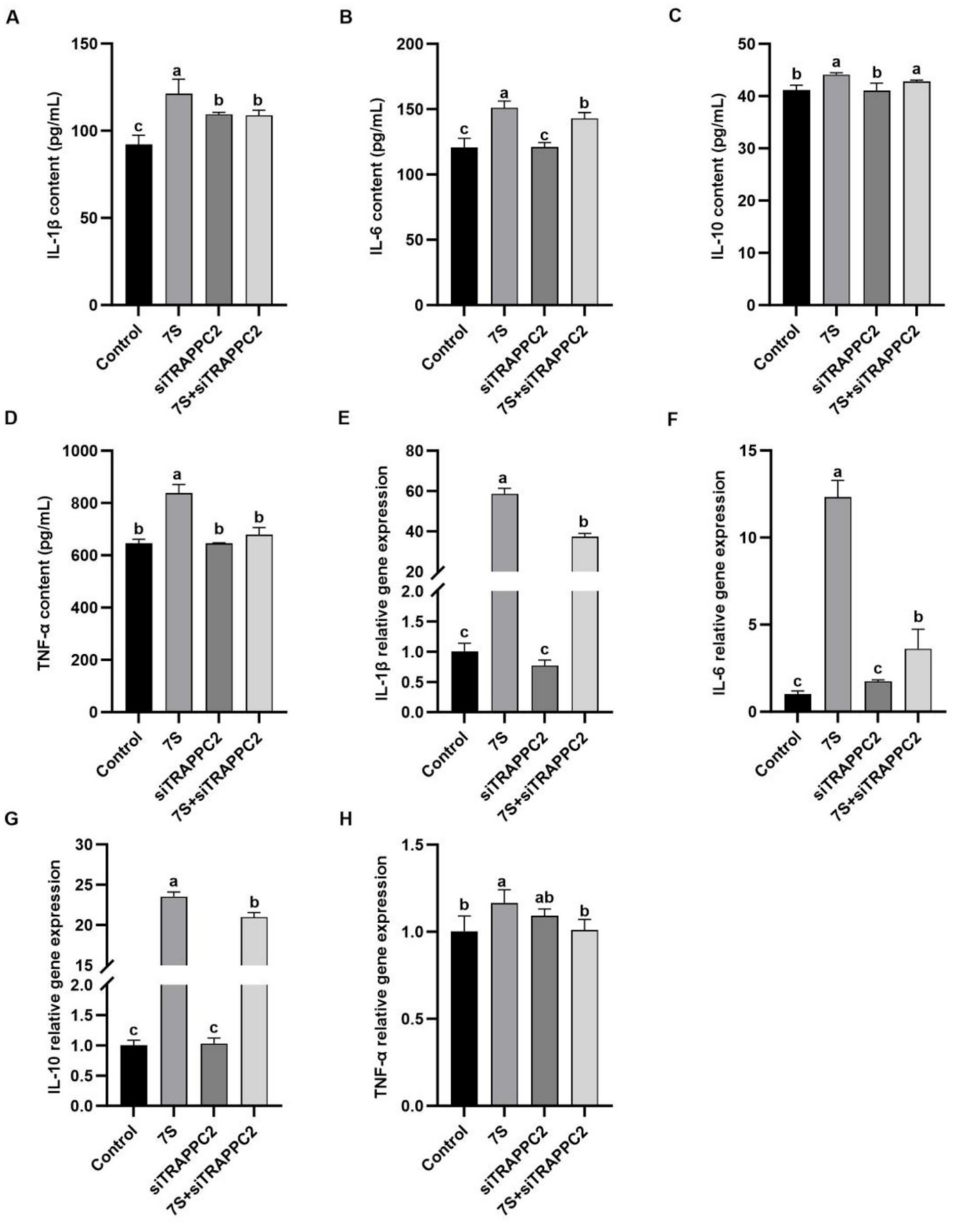
Effects of the cytokine levels of β-Conglycinin-induced RAW264.7 cells treated with effective TRAPPC2 siRNA. Control: untreated cells; 7S group: β-Conglycinin (7S)-treated cells; siTRAPPC2 group: siTRAPPC2-treated cells; 7S + siTRAPPC2 group: 7S + siTRAPPC2-treated cells. **(A–D)** The levels of inflammatory factor content (IL-1β, IL-6, IL-10, TNF-α) in the RAW264.7 cells supernatants. **(E–H)** The levels of inflammatory factor expression (IL-1β, IL-6, IL-10, TNF-α) in the RAW264.7 cells. ^a, b, c^*P* < 0.05.

In Figure 9, after 7S + CFP-siRNA transfection, the contents of IL-1β, IL-6, IL-10, and the relative mRNA expression of IL-1β, IL-6, and IL-10 in 7S + CFP-siRNA group were significantly lower than those in 7S treatment group (*P* < 0.05). In Figure 10, the levels of IL-1β, IL-6, TNF-α, and the relative mRNA expression of IL-1β, IL-6, and TNF-α in the 7S + TRAPPC2-siRNA treatment group were significantly lower than those in the 7S treatment group (*P* < 0.05). However, the levels of IL-6 and IL-10 and the relative mRNA expression levels of IL-1β, IL-6, and IL-10 in the 7S + TRAPPC2-siRNA/CFP-siRNA treatment group were still significantly higher than those in the control group (*P* < 0.05). In conclusion, CFP and TRAPPC2 genes could regulate the immune response of RAW264.7 cells induced by β-Conglycinin, but could not completely alleviate the immune injury induced by soybean antigen.

### 3.10 Effects of the cytokine levels of β-conglycinin-induced IPEC-J2 cells treated with effective CCL19 siRNA

**Figure 11:**
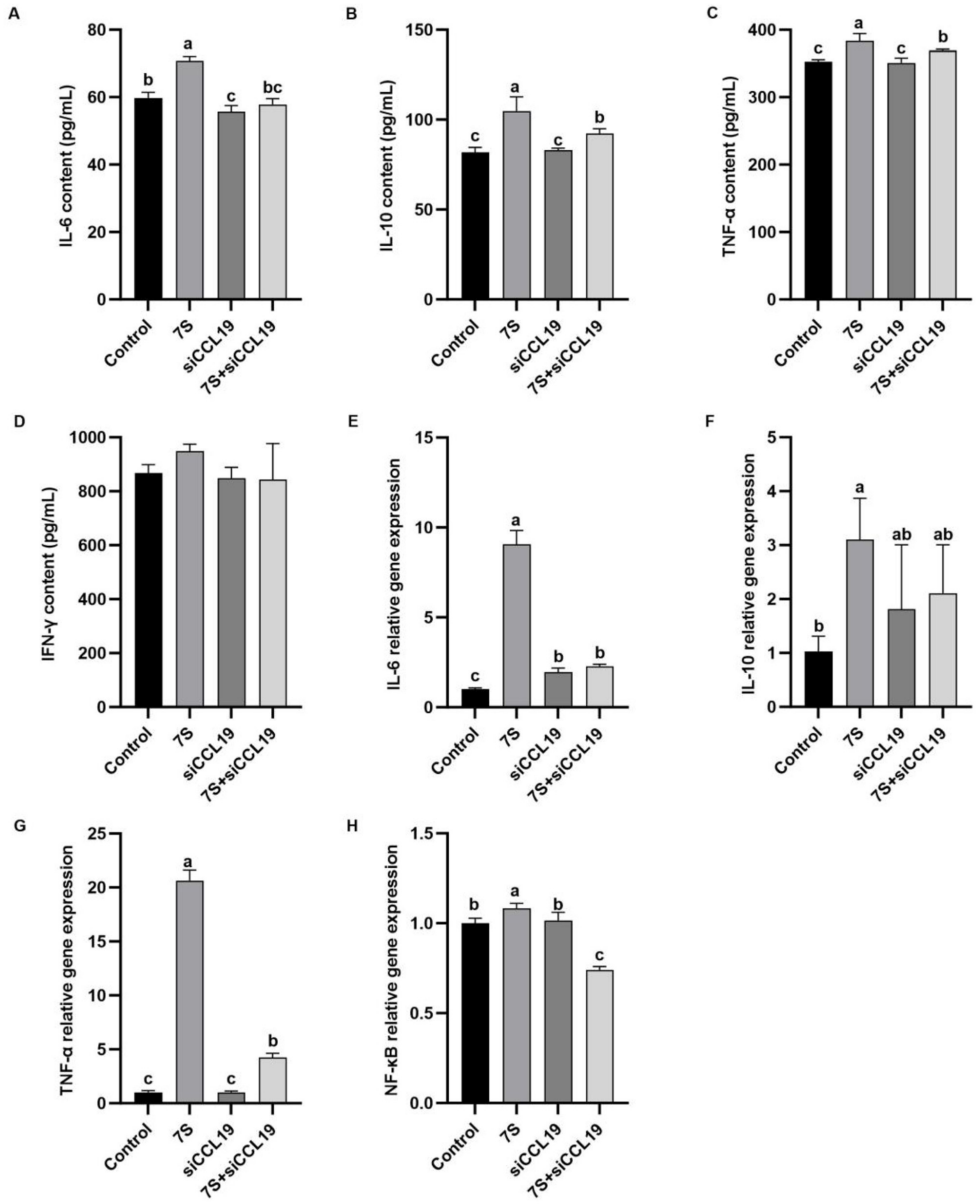
Effects of the cytokine levels of β-Conglycinin-induced IPEC-J2 cells treated with effective CCL19 siRNA. Control: untreated cells; 7S group: β-Conglycinin (7S)-treated cells; siCCL19 group: siCCL19-treated cells; 7S + siCCL19 group: 7S + siCCL19-treated cells. **(A–D)** The levels of inflammatory factor content (IL-6, IL-10, TNF-α, IFN-γ) in the IPEC-J2 cells supernatants. **(E–H)** The levels of inflammatory factor expression (IL-6, IL-10, TNF-α, NF-κB) in the IPEC-J2 cells. ^a, b, c^*P* < 0.05.

In Figure 11, *in vitro* experiments showed that IPEC-J2 cells transfected with specific CCL19-siRNA, The levels of TNF-α, IL-6, IL-10, and the relative mRNA expression of TNF-α, IL-6, and NF-κB in 7S + CCL19-siRNA treatment group were significantly lower than those in 7S treatment group (*P* < 0.05). However, compared with the control group, the 7S + CCL19-siRNA group still had significantly higher levels of IL-10 and TNF-α and relative mRNA expression of IL-6 and TNF-α, and significantly lower content and relative mRNA expression of NF-κB (*P* < 0.05). These results indicate that the CCL19 gene can regulate the inflammatory response of IPEC-J2 cells induced by β-Conglycinin, but cannot completely alleviate the immune inflammatory injury induced by soy antigen.

## 4 Discussion

Soybean is a valuable source of protein for both humans and animals, owing to its balanced amino acid composition and functional advantages (9). Nevertheless, soybean allergy is a severe health problem that occurs more frequently in early life (32). The skin sensitization test can be used to detect hypersensitivity reactions and immune status (33). Previously, Sun et al. found that feeding soybean antigen increased erythema area and diarrhea rate (25), which is similar to our experimental results, laying the groundwork for selecting allergic piglets for follow-up tests in the current study.

It was demonstrated in this study that the total serum IgG and IgE antibody levels were increased in the allergic group which was consistent with the findings from other studies (17, 25). The analysis of cytokines showed that piglets in the allergic group had higher IL-1β, IL-4, and TNF-α compared with the non-allergic group. The inflammation factor of IL-4 is secreted by Th2 cells, which stimulate the proliferation of B lymphocytes and generate IgE and IgG antibodies (17). IL-1β and TNF-α are mainly secreted by macrophages, activate NF-κB, increase intestinal epithelial permeability, destroy the intestinal tight junction barrier, and induce intestinal inflammation (34). Therefore, the soybean antigen-induced intestinal immune response of piglets is related to the upregulation of inflammatory factors, Th1/Th2 immune balance, and activation of NF-κB signaling pathway.

Epidemiological studies of food allergies have found that allergic reactions are not evenly distributed between men and women and with a higher risk in women (19, 20). Wang et al. also found that the female mice treated with ovalbumin (OVA) did exhibit more serious systemic anaphylaxis than male mice, and explored the potential reasons for gender differences in food allergy caused by estrogen (22). A harmful role for estrogen signaling in the setting of a food allergy is that an increased estrogen increases the level of immunoglobulin and impaired B cell function (35, 36), in addition, estrogen stimulates mast cells to release histamine and down-regulates the DAO enzyme that clears histamine, at the same time, histamine stimulates the ovaries to produce more estrogen (37, 38). Similarly, we also found that female piglets were more sensitive to soybean antigens than male piglets, and the specific SNPs of piglets in both allergic and non-allergic groups were mainly located in the X chromosome and the Estrogen signaling pathway had a significant change. Thus, the secretion of estrogen may be the reason for the strong sensitivity of female piglets to soybean antigens.

Nutrigenomics is the study of the interaction between nutrients and genes, encompassing genetics, nutrition, physiology, biochemistry, metabolomics, proteomics, and transcriptomics (39). PIR is called a paired immunoglobulin-like receptor, PIR-A is an activated receptor and PIR-B is an inhibitory receptor expressed on the surfaces of dendritic cells (DCs), B cells, mast cells, and macrophages. PIR-B inhibits Th1 cell response and induces Th2 cell differentiation cells, leading to the imbalance of Th1/Th2 cells. Some studies showed that the PIR-B disturbs the Th17/Treg balance to inhibit antitumor immunity (40–44). Complement factor properdin (CFP) plays an active role in regulating T cell immunity (45–47). It has been reported that mutations in the exon of the CFP gene on the X chromosome can cause meningitis (48). This finding is similar to our discovery that piglets with mutations in the CFP gene in the allergic group also exhibit symptoms of enteritis. The transfection of specific properdin siRNA can reduce properdin secretion by dendritic cells and limit the proliferation of T cells. Consequently, the secretion of TNF-α and IL-17 by T cells decreases (49). Similarly, our research indicates that silencing the CFP gene can reduce the secretion of IL-1β, IL-6, IL-8, and TNF-α levels induced by soybean antigens. Thus, the CFP gene may serve as a new therapeutic target for β-Conglycinin-induced immune injuries.

TRAPP is a protein that is important for secretory and endocytic pathways. TRAPPC2 is the main adaptor protein that helps the six core subunits of TRAPP interact with TRAPPC9 or TRAPPC8. If the TRAPPC2 protein is mutated, it can misfold and degrade, causing a loss of TRAPPC2 function. This means that it cannot interact with TRAPPC9 and TRAPPC8 (50), leading to a lack of immune response. Therefore, a mutation in the TRAPPC2 gene may be a reason for piglets being insensitive to soybean antigens. Additionally, inhibiting the expression of the TRAPPC2 gene can reduce the immune inflammatory damage caused by β-Conglycinin in RAW264.7 cells. However, there is almost no research on the SOWAHD gene, so we currently cannot know its relationship with soybean antigens. We have identified changes in amino acids within genes and changes in bases at mutation sites. These changes in amino acids are likely to affect the function and structure of genes, which in turn can impact the sensitivity of soybean antigens. Since we found that the expression abundance of PIR and SOWAHD genes in RAW264.7 cells was very low in our preliminary experiments, and the PCR technique was not easy to detect, it was difficult to carry out the next verification experiment. Therefore, we only selected CFP and TRAPPC2 genes with relatively high expression for further study. Based on our analysis, we believe that the TRAPPC2 and CFP genes may play a part in the differences in piglets' sensitivity to soybean antigens, but the roles of PIR and SOWAHD genes in soybean antigen-induced sensitivity differences in piglets remain to be verified.

Food allergy was once thought to be caused by Th1/Th2 imbalance (51), but later studies have shown IL-17 as a potential biological marker of food allergy (52, 53). IL-17C can increase the secretion of cytokines (TNF-α, IL-6, IL-1β), and chemokines (KC, CXCL2, CCL20) through the activation of NF-κB and MAPK signaling pathways (54, 55). In addition, chemokines are essential regulators of the immune response in the body. CCL19 can activate the PI3K/AKT signaling pathway, leading to the release of various inflammatory factors including NF-κB transcription factor. This can trigger an inflammatory response and also interact with CCL21 to regulate the Th1/Th2 immune balance (56). Studies have shown that inhibiting CCL21 expression can help alleviate clinical symptoms and intestinal tissue damage in mice with ulcerative colitis (UC) (57). Therefore, we chose to further verify the role of the CCL19 gene in soybean antigen-induced intestinal injury in piglets. Similarly, treating IPEC-J2 cells with CCL19 siRNA has also demonstrated that CCL19 plays an important role in regulating soybean antigen-induced intestinal inflammation.

Amino acids can indirectly regulate the intestinal immune response (58). Arginine and glutamine are key mediators of mTORC1 activation, which regulate key metabolic pathways in the intestinal barrier and have beneficial effects on the gut-related immune system (59). It was suggested that tryptophan metabolism plays an important role in allergic diseases, and tryptophan is a precursor to the synthesis of serotonin (5-hydroxytryptamine, 5-HT) and melatonin (n-acetyl-5-methoxytryptamine) (60). Tryptophan (Trp) is capable of producing a variety of aryl hydrocarbon receptor (AhR) ligands through several metabolic pathways (61). It was found that AhR inhibits IL-17-mediated inflammatory response through negative feedback loops, thus inhibiting the occurrence and development of Ulcerative colitis (UC) (62), and the activation of the AHR can suppress allergic sensitization by suppressing the absolute number of precursor and effector T cells and affecting DCs (63). Similarly, our results demonstrated that the contents of phenylalanine, tryptophan, leucine, arginine, valine, and glutamine in the intestinal metabolites of piglets in the allergic group were significantly increased, implying that soybean antigen affected the metabolism and utilization of amino acid in the intestine of piglets, caused the intestinal immune response of piglets, and finally induced the allergy.

## 5 Conclusion

In conclusion, there are individual differences in the allergic response of weanling piglets to soy antigens, and the rates of allergy show large differences between the genders. Meanwhile, the differentially expressed candidate genes PIR, CFP, TRAPPC2, SOWAHD, and CCL19 were found to be induced by soybean antigens in piglets. The genetic mechanism of soybean antigen-induced allergy in piglets was preliminarily revealed, and the intestinal damage of piglets induced by soybean antigen mainly affected the utilization and metabolism of intestinal nutrients, especially the digestion and absorption of protein and the synthesis and metabolism of amino acids. This study provides a scientific reference for the differential feeding, precision nutrition, disease prevention, and control of piglets, of piglets, and provides a theoretical basis for the study of the anti-nutritional mechanism of soybean antigen and lays a foundation for human foodborne diseases.

## Data Availability

The datasets presented in this study can be found in online repositories. The names of the repository/repositories and accession number(s) can be found in the article/Supplementary material.

## References

[1] Hao Y Zhan Z Guo P Piao X Li D. Soybean β-conglycinin-induced gut hypersensitivity reaction in a piglet model. Arch Animal Nutr. (2009) 63:188–202. 10.1080/17450390902860026

[2] Yi D Hou Y Mei H Wang L Hu CA Wu G. beta-Conglycinin enhances autophagy in porcine enterocytes. Amino Acids. (2017) 49:203–7. 10.1007/s00726-016-2352-7 27761755

[3] Zhao Y Qin GX Sun ZW Zhang B Wang T. Effects of glycinin and β-conglycinin on enterocyte apoptosis, proliferation and migration of piglets. Food Agric Immunol. (2010) 21:209–18. 10.1080/09540101003596644

[4] Peng C Ding X Zhu L He M Shu Y Zhang Y. beta-Conglycinin-induced intestinal porcine epithelial cell damage via the nuclear factor kappaB/mitogen-activated protein kinase signaling pathway. J Agric Food Chem. (2019) 67:9009–21. 10.1021/acs.jafc.9b02784 31319030

[5] Zhang W Tan B Ye G Wang J Dong X Yang Q. Identification of potential biomarkers for soybean meal-induced enteritis in juvenile pearl gentian grouper, Epinephelus lanceolatus♂ × Epinephelus fuscoguttatus♀. Aquaculture. (2019) 512:734337. 10.1016/j.aquaculture.2019.734337

[6] Wang S. *Lactobacillus rhamnosus* GG reduces β-conglycinin-allergy-induced apoptotic cells by regulating bacteroides and bile secretion pathway in intestinal contents of BALB/c mice. Nutrients. (2020) 13:55. 10.3390/nu13010055 33375432 PMC7823992

[7] Peng C Cao C He M Shu Y Tang X Wang Y. Soybean glycinin- and beta-conglycinin-induced intestinal damage in piglets via the p38/JNK/NF-kappaB signaling pathway. J Agric Food Chem. (2018) 66:9534–41. 10.1021/acs.jafc.8b03641 30139257

[8] Stark KG Falkowski NR Brown CA McDonald RA Huffnagle GB. Contribution of the microbiome, environment, and genetics to mucosal type 2 immunity and anaphylaxis in a murine food allergy model. Front Allergy. (2022) 3:851993. 10.3389/falgy.2022.851993 35769569 PMC9234882

[9] Zheng S Yin S Qin G Yao J Liu S Han J. Gastrointestinal digestion and absorption of soybean beta-conglycinin in an early weaned piglet model: an initial step to the induction of soybean allergy. Food Chem. (2023) 427:136640. 10.1016/j.foodchem.2023.136640 37429130

[10] Kilshaw PJ Sissons JW. Gastrointestinal allergy to soyabean protein in preruminant calves allergenic constituents of soyabean products. Res Vet Sci. (1979) 27:366–71. 10.1016/S0034-5288(18)32809-1 575571

[11] Liu X Fu Y Wang J Wu D Li S Wang C. beta-Conglycinin induces the formation of neutrophil extracellular traps dependent on NADPH oxidase-derived ROS, PAD4, ERK1/2 and p38 signaling pathways in mice. Food Funct. (2021) 12:154–61. 10.1039/D0FO02337J 33289753

[12] Zhao Y Naren G Qiang J Qin G Bao N Farouk MH. Identification of allergic epitopes of soybean beta-conglycinin in different animal species. Front Vet Sci. (2020) 7:599546. 10.3389/fvets.2020.599546 33490132 PMC7820328

[13] Qin G. Processing soybeans of different origins. Response of a Chinese and a Western pig breed to dietary inclusion (1996). 10.18174/210486

[14] Marenholz I Grosche S Kalb B Rüschendorf F Blümchen K Schlags R. Genome-wide association study identifies the SERPINB gene cluster as a susceptibility locus for food allergy. Nat Commun. (2017) 8:1056. 10.1038/s41467-017-01220-0 29051540 PMC5648765

[15] Crespo-Piazuelo D Migura-Garcia L Estellé J Criado-Mesas L Revilla M Castelló A. Association between the pig genome and its gut microbiota composition. Sci Rep. (2019) 9:8791. 10.1038/s41598-019-45066-6 31217427 PMC6584621

[16] Neefjes J Jongsma ML Paul P Bakke O. Towards a systems understanding of MHC class I and MHC class II antigen presentation. Nat Rev Immunol. (2011) 11:823–36. 10.1038/nri3084 22076556

[17] Chang M Zhao Y Qin G Zhang X. Fructo-oligosaccharide alleviates soybean-induced anaphylaxis in piglets by modulating gut microbes. Front Microbiol. (2018) 9:2769. 10.3389/fmicb.2018.02769 30524396 PMC6256172

[18] Johnston A Fritz Y Dawes SM Diaconu D Al-Attar PM Guzman AM. Keratinocyte overexpression of IL-17C promotes psoriasiform skin inflammation. J Immunol. (2013) 190:2252–62. 10.4049/jimmunol.1201505 23359500 PMC3577967

[19] DunnGalvin A Hourihane JO Frewer L Knibb RC Oude Elberink JN Klinge I. Incorporating a gender dimension in food allergy research: a review. Allergy. (2006) 61:1336–43. 10.1111/j.1398-9995.2006.01181.x 17002711

[20] Pali-Scholl I Jensen-Jarolim E. Gender aspects in food allergy. Curr Opin Allergy Clin Immunol. (2019) 19:249–55. 10.1097/ACI.0000000000000529 30893085

[21] Surman SL Jones BG Penkert RR Sealy RE Marion T Thomas PG. How estrogen, testosterone, and sex differences influence serum immunoglobulin isotype patterns in mice and humans. Viruses. (2023) 15:482. 10.3390/v15020482 36851695 PMC9961480

[22] Wang J Guo X Chen C Sun S Liu G Liu M. Gender differences in food allergy depend on the PPAR gamma/NF-kappaB in the intestines of mice. Life Sci. (2021) 278:119606. 10.1016/j.lfs.2021.119606 33974930

[23] Cabanillas B Jappe U Novak N. Allergy to peanut, soybean, and other legumes: recent advances in allergen characterization, stability to processing and IgE cross-reactivity. Mol Nutr Food Res. (2018) 62:446. 10.1002/mnfr.201700446 28944625

[24] Zhao Y Qin G Sun Z Zhang X Bao N Wang T. Disappearance of immunoreactive glycinin and beta-conglycinin in the digestive tract of piglets. Arch Anim Nutr. (2008) 62:322–30. 10.1080/17450390802190318 18763626

[25] Sun P Li D Dong B Qiao S Ma X. Effects of soybean glycinin on performance and immune function in early weaned pigs. Arch Anim Nutr. (2008) 62:313–21. 10.1080/17450390802066419 18763625

[26] Li H Durbin R. Fast and accurate short read alignment with Burrows-Wheeler transform. Bioinformatics. (2009) 25:1754–60. 10.1093/bioinformatics/btp324 19451168 PMC2705234

[27] McKernan KJ Peckham HE Costa GL McLaughlin SF Fu Y Tsung EF. Sequence and structural variation in a human genome uncovered by short-read, massively parallel ligation sequencing using two-base encoding. Genome Res. (2009) 19:1527–41. 10.1101/gr.091868.109 19546169 PMC2752135

[28] Wang K Li M Hakonarson H. ANNOVAR functional annotation of genetic variants from high-throughput sequencing data. Nucleic Acids Res. (2010) 38:e164. 10.1093/nar/gkq603 20601685 PMC2938201

[29] Su XL Zhao SS Xu WJ Shuang L Zheng GD Zou SM. Efficiently whole-genomic mutagenesis approach by ARTP in blunt snout bream (*Megalobrama amblycephala*). Aquaculture. (2022) 555:738241. 10.1016/j.aquaculture.2022.738241

[30] Mi M Chang M Huang Y Zhao J Pan L Bao N. Fructo-oligosaccharides ameliorate intestinal mechanical barrier injury in piglets induced by soybean antigen *in vitro* and *in vivo*. Curr Protein Pept Sci. (2023) 24:267–76. 10.2174/1389203724666230224090312 36825707

[31] Yi G Li H Liu M Ying Z Zhang J. Liu X. Soybean protein-derived peptides inhibit inflammation in LPS-induced RAW2647 macrophages via the suppression of TLR4-mediated MAPK-JNK and NF-kappa B activation. J Food Biochem. (2020) 44:e13289. 10.1111/jfbc.13289 32537742

[32] Nordlee J Taylor SL Jones RT Yunginger JW. Allergenicity of various peanut products as determined by RAST inhibition. J Allergy Clin Immunol. (1981) 68:376–82. 10.1016/0091-6749(81)90136-6 7299002

[33] Helm RM Furuta GT Stanley JS Ye J Cockrell G Connaughton C. A neonatal swine model for peanut allergy. J Allergy Clin Immunol. (2002) 109:136–42. 10.1067/mai.2002.120551 11799380

[34] Kaminsky LW Al-Sadi R Ma TY. IL-1beta and the intestinal epithelial tight junction barrier. Front Immunol. (2021) 12:767456. 10.3389/fimmu.2021.767456 34759934 PMC8574155

[35] Bonds RS Midoro-Horiuti T. Estrogen effects in allergy and asthma. Curr Opin Allergy Clin Immunol. (2013) 13:92–9. 10.1097/ACI.0b013e32835a6dd6 23090385 PMC3537328

[36] Aguilar-Pimentel JA Cho YL Gerlini R Calzada-Wack J Wimmer M Mayer-Kuckuk P. Increased estrogen to androgen ratio enhances immunoglobulin levels and impairs B cell function in male mice. Sci Rep. (2020) 10:18334. 10.1038/s41598-020-75059-9 33110090 PMC7591566

[37] Guo TL. (Xeno)estrogen regulation of food allergy. J Immunotoxicol. (2008) 5:259–70. 10.1080/15376510802312290 18830886

[38] Munoz-Cruz S Mendoza-Rodríguez Y Nava-Castro KE Yepez-Mulia L Morales-Montor J. Gender-related effects of sex steroids on histamine release and FcepsilonRI expression in rat peritoneal mast cells. J Immunol Res. (2015) 2015:351829. 10.1155/2015/351829 25973435 PMC4417946

[39] Kiani AK Bonetti G Donato K Kaftalli J Herbst KL Stuppia L. Polymorphisms, diet and nutrigenomics. J Prev Med Hyg. (2022) 63:E125–41. 10.15167/2421-4248/jpmh2022.63.2S3.2754 PMC9710387 36479483

[40] Takai T. A novel recognition system for MHC class I molecules constituted by PIR. Adv Immunol. (2005) 88:161–92. 10.1016/S0065-2776(05)88005-8 16227090

[41] Rothe K Quandt D Köhler G Jasinski-Bergner S Seliger B Pierer M. PIR-B expressing CD8+ T cells exhibit features of Tc1 and Tc17 in SKG mice. Rheumatology (Oxford). (2019) 58:2325–9. 10.1093/rheumatology/kez256 31257448

[42] Wu H Zheng X Dong L Li C Zhang M Wang G. Pir-B inhibits the DC function and disturbs the Th17/Treg balance in lung cancer murine model. Oncotarget. (2017) 8:114710–21. 10.18632/oncotarget.21763 29383114 PMC5777726

[43] Munitz A Cole ET Beichler A Groschwitz K Ahrens R Steinbrecher K. Paired immunoglobulin-like receptor B (PIR-B) negatively regulates macrophage activation in experimental colitis. Gastroenterology. (2010) 139:530–41. 10.1053/j.gastro.2010.04.006 20398663 PMC3423916

[44] Uddin J Tomar S Sharma A Waggoner L Ganesan V Marella S. PIR-B regulates CD4(+) IL17a(+) T-cell survival and restricts T-cell-dependent intestinal inflammatory responses. Cell Mol Gastroenterol Hepatol. (2021) 12:1479–502. 10.1016/j.jcmgh.2021.06.013 34242819 PMC8531983

[45] Narni-Mancinelli E Gauthier L Baratin M Guia S Fenis A Deghmane AE. Complement factor P is a ligand for the natural killer cell-activating receptor NKp46. Sci Immunol. (2017) 2:eaam9628. 10.1126/sciimmunol.aam9628 28480349 PMC5419422

[46] Cui G Geng L Zhu L Lin Z Liu X Miao Z. CFP is a prognostic biomarker and correlated with immune infiltrates in gastric cancer and lung cancer. J Cancer. (2021) 12:3378–90. 10.7150/jca.50832 33976747 PMC8100816

[47] Dixon KO O'Flynn J Klar-Mohamad N Daha MR van Kooten C. Properdin and factor H production by human dendritic cells modulates their T-cell stimulatory capacity and is regulated by IFN-gamma. Eur J Immunol. (2017) 47:470–80. 10.1002/eji.201646703 28105653 PMC5363362

[48] Seitsonen S Helminen M Jarva H Meri S Järvelä I. [Properdin mutations a risk factor for meningitis]. Duodecim. (2010) 126:1071–5. 20593630

[49] van Essen MF Schlagwein N van Gijlswijk-Janssen DJ Ruben JM van Kooten C. Properdin produced by dendritic cells contributes to the activation of T cells. Immunobiology. (2022) 227:152246. 10.1016/j.imbio.2022.152246 35843030

[50] Zong M Wu XG Chan CW Choi MY Chan HC Tanner JA. The adaptor function of TRAPPC2 in mammalian TRAPPs explains TRAPPC2-associated SEDT and TRAPPC9-associated congenital intellectual disability. PLoS ONE. (2011) 6:e23350. 10.1371/journal.pone.0023350 21858081 PMC3156116

[51] Han S Sun L He F Che H. Anti-allergic activity of glycyrrhizic acid on IgE-mediated allergic reaction by regulation of allergy-related immune cells. Sci Rep. (2017) 7:7222. 10.1038/s41598-017-07833-1 28775294 PMC5543155

[52] Song X He X Li X Qian Y. The roles and functional mechanisms of interleukin-17 family cytokines in mucosal immunity. Cell Mol Immunol. (2016) 13:418–31. 10.1038/cmi.2015.105 27018218 PMC4947810

[53] Berin MC Lozano-Ojalvo D Agashe C Baker MG Bird JA Nowak-Wegrzyn A. Acute FPIES reactions are associated with an IL-17 inflammatory signature. J Allergy Clin Immunol. (2021) 148:895–901.e6. 10.1016/j.jaci.2021.04.012 33891982 PMC8675150

[54] Song X Zhu S Shi P Liu Y Shi Y Levin SD. IL-17RE is the functional receptor for IL-17C and mediates mucosal immunity to infection with intestinal pathogens. Nat Immunol. (2011) 12:1151–8. 10.1038/ni.2155 21993849

[55] Huang J Meng S Hong S Lin X Jin W Dong C. IL-17C is required for lethal inflammation during systemic fungal infection. Cell Mol Immunol. (2016) 13:474–83. 10.1038/cmi.2015.56 26166766 PMC4947823

[56] Zhang JF Li Y Zhang AZ He QQ Du YC Cao W. Expression and pathological significance of CC chemokine receptor 7 and its ligands in the airway of asthmatic rats exposed to cigarette smoke. J Thorac Dis. (2018) 10:5459–67. 10.21037/jtd.2018.08.124 30416795 PMC6196219

[57] Zhang H Zhang X Ding X Cao W Qu L Zhou G. Effect of secondary lymphoid tissue chemokine suppression on experimental ulcerative colitis in mice. Genet Mol Res. (2014) 13:3337–45. 10.4238/2014.April.29.12 24841666

[58] Zhao-Lai D. Amino acid metabolism in intestinal bacteria: links between gut ecology and host health. Front Biosci. (2011) 16:1768. 10.2741/3820 21196263

[59] Carroll B Maetzel D Maddocks OD Otten G Ratcliff M Smith GR. Correction: control of TSC2-Rheb signaling axis by arginine regulates mTORC1 activity. Elife. (2020) 9:65744. 10.7554/eLife.65744 PMC7744091 33325825

[60] von Bubnoff D Bieber T. The indoleamine 2,3-dioxygenase (IDO) pathway controls allergy. Allergy. (2012) 67:718–25. 10.1111/j.1398-9995.2012.02830.x 22519427

[61] Sun M Ma N He T Johnston LJ Ma X. Tryptophan (Trp) modulates gut homeostasis via aryl hydrocarbon receptor (AhR). Crit Rev Food Sci Nutr. (2020) 60:1760–8. 10.1080/10408398.2019.1598334 30924357

[62] Chng SH Kundu P Dominguez-Brauer C Teo WL Kawajiri K Fujii-Kuriyama Y. Ablating the aryl hydrocarbon receptor (AhR) in CD11c+ cells perturbs intestinal epithelium development and intestinal immunity. Sci Rep. (2016) 6:23820. 10.1038/srep23820 27068235 PMC4828637

[63] Schulz VJ Smit JJ Pieters RH. The aryl hydrocarbon receptor and food allergy. Vet Q. (2013) 33:94–107. 10.1080/01652176.2013.804229 23745732

